# Rheological Properties of Soft Waste Granulates Produced in the Fabrication Process of Ceramic Tiles and the Possibility of Their Reuse

**DOI:** 10.3390/ma15041366

**Published:** 2022-02-12

**Authors:** Łukasz Wójcik, Marcin Gajek, Alicja Rapacz-Kmita, Joanna Mastalska-Popławska, Robert Pacan, Izabela Puchyrska, Piotr Sacha, Dawid Cegłowski

**Affiliations:** 1 Faculty of Materials Science and Ceramics, AGH University of Science and Technology, Mickiewicza 30, 30-059 Krakow, Poland; mgajek@agh.edu.pl (M.G.); kmita@agh.edu.pl (A.R.-K.); jmast@agh.edu.pl (J.M.-P.); 2 Cerrad Sp. z o.o., ul. Radomska 49B, 27-200 Starachowice, Poland; r.pacan@cerrad.com (R.P.); i.puchyrska@cerrad.com (I.P.); p.sacha@cerrad.com (P.S.); d.ceglowski@cerrad.com (D.C.)

**Keywords:** soft waste granulate, powder flow, rheology, ceramic

## Abstract

The paper presents the results of research on the influence of the granulometric composition on the rheological properties of granulates from soft waste produced in the manufacturing of ceramic tiles using the Lamgea and Continua methods in terms of the possibility of their reuse. The composition of the granulates was modified by removing individual grain fractions in three measurement series. Comparatively, the measurement samples for the production granulates were prepared in the same way. Microscopic observations and granulometric analysis showed significant differences in the grain shape and grain size distribution of granulates. The soft waste granulates also showed much worse flow ability than the production granulates. It was shown that the removal of the smallest fractions significantly improved the rheological properties of soft waste granulates. This tendency was also observed in the case of measurements of changes in the bulk density. A Brookfield powder analyzer was used for rheological tests, and a flow analysis was performed using the numerical Jenike classification.

## 1. Introduction

The production of ceramic tiles has recently undergone several significant changes in the technological process. Starting from the introduction of fast firing, through the replacement of traditional methods of decorating with digital printing, to the introduction of new methods, such as continuous strand using the “Continua” method and forming large slabs using the “Lamgea” method. In both “pressing” technologies, the final size of the tiles is shaped by cutting the unfired strand or large-size slab [1,2,3]. Any major change in the production process usually entails adjustments to the rest of the production steps. In the case of the Continua and Lamgea methods, there was a problem with the management of large amounts of soft waste, (up to 30%), generated when cutting the desired tile formats.

This waste, being a full-value production mass, can be reused in the production process, but requires reprocessing. Returning waste to the spray dryer would be associated with high energy expenditure, therefore other, more effective methods of using it are sought [4,5]. One such method may be the production of granulates by mechanical grinding to obtain the appropriate particle size.

The obtained re-granulate from soft waste (crushed green body granulate) differs significantly from the granulates produced in spray dryers [6,7], as it has a different grain shape, grain size, and rheological properties. The rheological properties of granulates for the production of ceramic tiles are particularly important due to the stages of the production process [1]. After production, the granulate is transported to silos, where it is homogenized and where it is self-compacted, which influences its flow and may lead to the formation of rathole flow, and even stop the flow due to the arching phenomenon [8,9]. Finally, the granulate is subjected to a compacting process, in which the degree of filling the mold and its initial packing depend on its rheological parameters. Therefore, the control of rheological properties of granulates is particularly important when granulates are produced by various methods, which may lead to significant differences in their behavior [6].

Research on the rheological properties of powders and granulates can be carried out in several ways, starting from the simplest, i.e., measuring the mass flow rate through the Ford cup or geometrically evaluated static angle of fall [10,11], to much more sophisticated ones, in which rheometers operating at controlled shear rate (CR) are used [12].

The aim of this study was to investigate the possibility of controlling the rheological properties of soft waste granulates through a relatively inexpensive operation consisting of the removal of individual grain fractions, so that the obtained granulate was as rheologically similar as possible to the production granulate. This operation would allow the soft waste granulate to be mixed with the production granulate.

Depending on the cutting format, the share of soft waste reaches up to 30% of production, which, for large plants, amounts to several thousand tons of waste per month. The possibility to significantly reduce the amount of material returned to the spray dryer would not only reduce production costs, but equally importantly, would significantly reduce energy consumption and, consequently, CO_2_ emissions.

The PFT device from Brookfield, based on the ring shear tester, was used for rheological tests of granulates [13]. In this system, the test sample is placed in the rotating lower ring and the consolidation stress is exerted by the upper ring. This system allows the determination of the entire flow function of the powder/granulate with a single measurement, i.e., the wall friction angle and the change in bulk density as a function of consolidation stress.

## 2. Materials and Methods

Materials provided by Cerrad Sp. z o.o. were used for the tests, i.e., production granulates produced in a spray dryer and used in current manufacturing processes as well as soft waste produced when cutting large-format boards pressed from production granulate in the SYSTEM LAMGEA 44000 technology by SYSTEM CERAMIC: Fiorano Modenese, Italy. The moisture content of the production granulate (PG) and the soft waste granulate (SG) was 5.4% and 5.2%, respectively. Samples of soft waste were taken from the production line and, after initial grinding to 5-15 cm, they were crushed in a laboratory jaw crusher LAB-02-130 manufactured by EKOLAB: Brzesko, Poland, with a jaw clearance of 5 mm. After this process, the resulting granulates were sieved through a 2 mm sieve.

The granulometric composition and grain shapes of soft waste granulates differ significantly from the parameters of production granulates manufactured in a spray dryer. Therefore, in order to investigate the influence of individual granulometric fractions on its rheological properties, the composition of the granulates was modified by removing individual grain fractions. In this way, three series of measurements were obtained. In the first one, the finest fraction was removed; in the second, the finest and coarsest was removed; and in the third, only the coarsest was removed. The same was done with the production granulate; dry sieving was conducted through a sieve set with the following sieve sizes: 1, 0.71, 0.60, 0.50, 0.425, 0.3, 0.25, 0.18, and 0.125 mm.

Microscopic observations of the separated granulometric fractions were made using the AZ100 optical microscope manufactured by NIKON: Tokyo, Japan. For each fraction the bulk density and angle of fall tests were carried out.

The rheological measurements of the flow function and changes of the bulk density as a function of consolidation stress were carried out using the PFT powder tester manufactured by Brookfield/Amtek: Middleboro, MA, USA. A measurement system with a diameter of 6 inches was used, and the measurements were carried out in the range up to 10 kPa.

**Figure 1:**
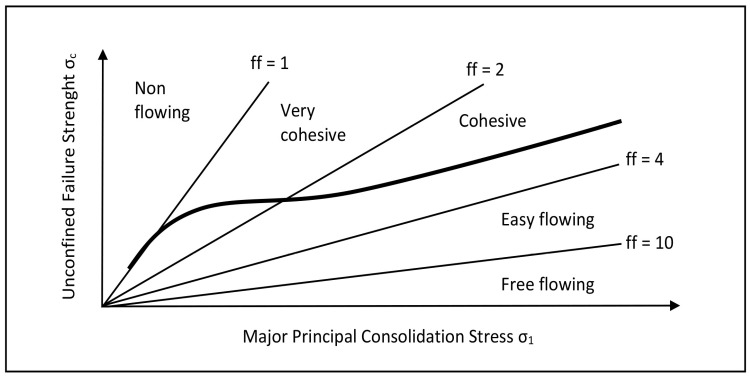
Graphical interpretation of the flow function using Jenike numerical classification [14].

On the basis of the flow function, the tested granulates can be easily classified using the Jenike classification [14], based on the consolidation ratio ff—the ratio of consolidation stress, σ_1_, and unlimited boundary stress, σ_c_ [15]. By drawing straight lines with a defined flow on the flow function graphs (Figure 1), the tested granulates can be directly assigned to one of five flow types: non-flowing, very cohesive, cohesive, easy flowing, and free flowing, corresponding to the behavior of granulates during flow. In the case of granulation of powders, one of the goals of this process is to improve their fluidity, thanks to which they are easier to transport, and most importantly, they fill the molds better, which facilitates the process of forming products.

## 3. Results and Discussion

### 3.1. Powder Characteristic

Granulates made of soft waste (SG) quite significantly differ in terms of grain size distribution compared to production granulates (PG) produced in spray dryers (Table 1). The greatest differences are seen in the coarsest (25.5% to 1.9% for fractions above 1 mm) and the finest (17.8% to 1.2% for fractions below 0.125 mm) fractions, but there are also differences in other fractions. Matching the grain curve of soft waste granulate to that of the production granulate would be quite troublesome in industrial conditions and would involve large losses of material. From the point of view of the production process, a much simpler method is the elimination of specific grain fractions from the initial granulate.

For the purposes of the study, three series of samples were prepared for each of the granulates. In the first series, successive fine fractions ranging from 0.125 to 0.425 mm were removed. In the second series, fine fractions ranging from 0.125 to 0.250 mm and the coarsest fractions 0.71 to 1 mm were separated. In the third series, coarse fractions ranging from 0.60 to 1 mm were separated. The detailed particle size distribution of the granulates obtained in this way is presented in Table 1 and Table 2.

Standard tests of bulk density and angle of fall were carried out for all prepared granulates, and the results are presented in Table 3.

**Figure 2:**
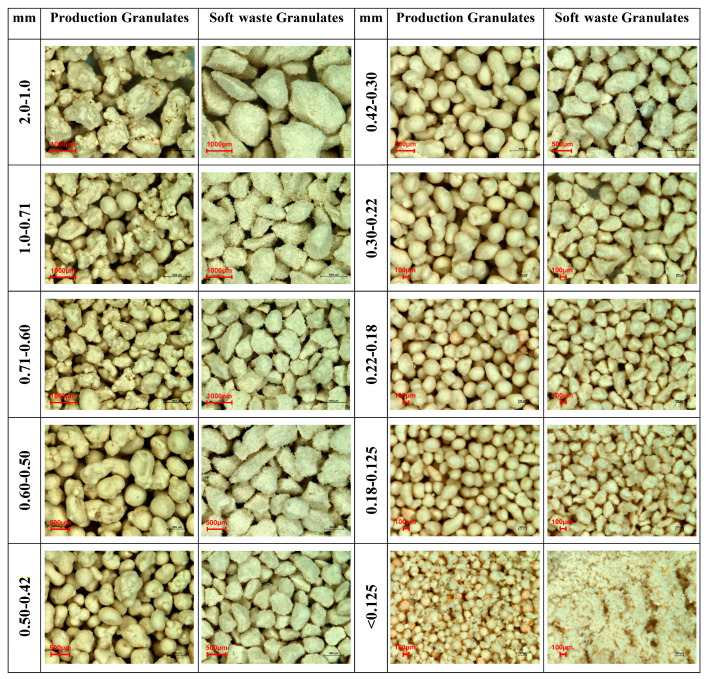
Images of grain fractions of production granulates and granulates from soft waste.

The photographs in Figure 2 present comparative images of the separated granulometric fractions.

The microscopic observations showed, as expected, differences between the granulate obtained in the spray dryer (the so-called standard) and the granulate obtained from grinding soft waste (pressed production waste).

In the case of production granulates, numerous agglomerates consisting of smaller grains are observed for fractions above 0.6 mm. For fractions in the range of 0.06–0.5 mm, both undefected grains and agglomerates are observed, while well-formed grains with spherical shapes are observed below 0.5 mm. The smallest grain fraction below 0.125 mm, apart from clearly visible spherical grains, also contains a small amount of dust fraction.

The microscopic images of granulates ranging from 2.0 to 0.125 mm obtained from soft waste shows grains with irregular, sharp-edged shapes, while moving on to the finer fractions, grains with an increasingly spherical, isometric shape are observed, whereas the smallest fraction below 0.125 mm were very dusty.

Bulk density measurements show quite marked differences between soft waste granulates and production granulates, with SG granulates generally exhibiting a lower bulk density and a greater angle of fall than their PG counterparts. This is related to a greater deviation from the spherical shape of these SG granulates, which in turn translates into their poorer packing in space and worse flow, and thus the measured higher values of the angle of fall. The recorded angles of fall differ only slightly between the samples within the series, and these changes, apart from the improvement of the flow after cutting off the finest fraction, do not show any major regularities.

### 3.2. Rheological Behavior

**Figure 3:**
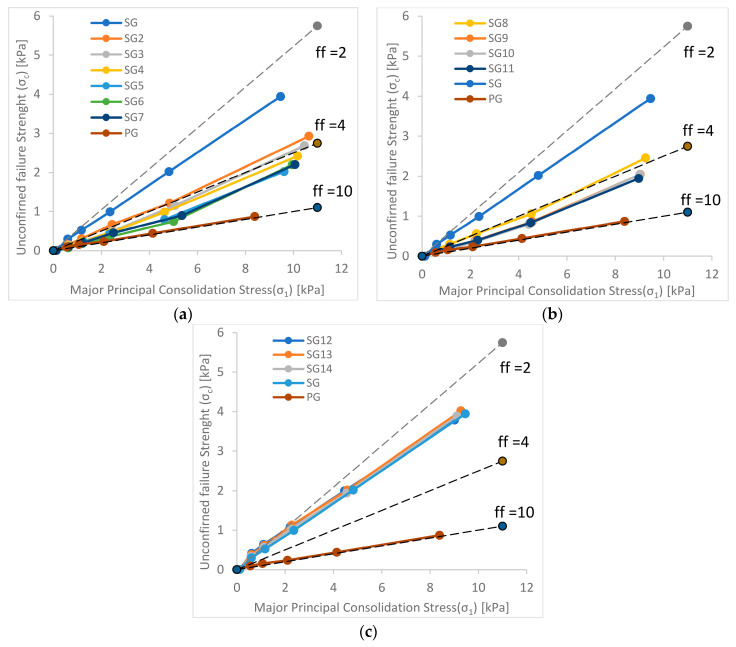
Flow function of soft waste granulates (**a**) series 1; (**b**) series 2; (**c**) series 3.

**Figure 4:**
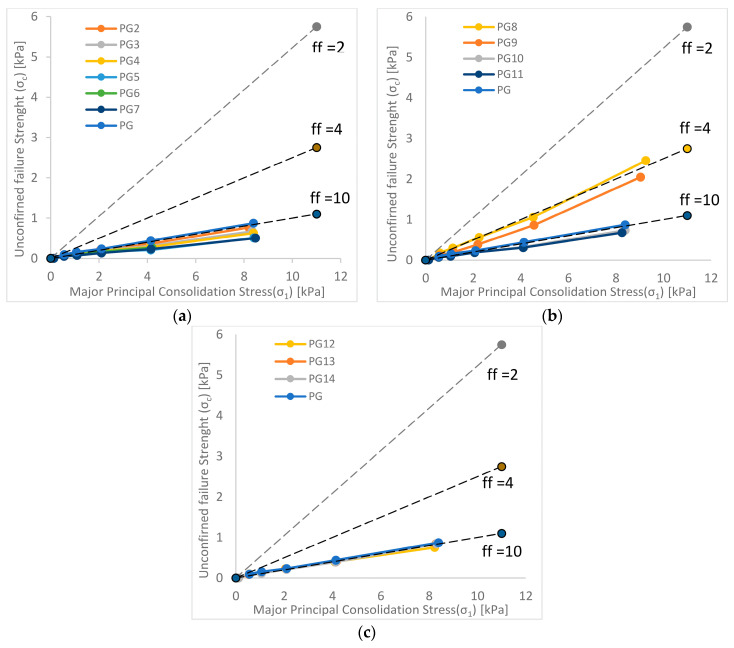
Flow function of production granulates (**a**) series 1; (**b**) series 2; (**c**) series 3.

Measurements for both granulates were carried out in three series, and their results, in the form of a flow function, together with Jenike’s graphical interpretation, are presented in Figure 3 and Figure 4. For soft waste granulate, the graphs additionally show the results recorded for the production granulate without modification (PG).

The results of the conducted research indicate a significantly worse flow of granulates from soft waste compared to granulates produced in a spray dryer. All the flow functions recorded for soft waste granulates are above the flow function of the production granulates (PG), which is on the border of the easy-flowing and free-flowing areas (consolidation factor close to 10).

The course of the flow function of granulate from soft waste (SG) places this material in the range of cohesive flows-consolidation factor ff [14] in the range of 2–4. However, changes in the particle size distribution shift the flow functions towards better flowability.

The greatest changes in the flowability of granulates from soft waste were observed in the case of series 1 and 2, i.e., for the first series (Figure 3a), a large jump in flow ability was observed after the removal of the finest fraction (0 to 0.180 mm), while the subsequent changes are less pronounced. For samples 4–7, a transition from the cohesive to the easily flowing area was observed, and further cutting of the lower fractions in the range of 0.30–0.425 mm did not change the nature of the flow of granulates, means that the recorded courses of the flow function looked similar.

In the case of series 2 (Figure 3b), in which the finest fractions were cut off (from the bottom) together with the simultaneous removal of the coarsest fractions, the greatest jump in rheological properties was observed for the first two samples, in which the bottom fractions were cut off in the range of 0–0.18 mm and above 1.0 mm, but the further cutting of the fine fraction had no effect on the flow function. For this series, similarly to series 1, a transition from the cohesive area to the easy-flowing area was observed for samples 9–11.

In the case of series 3 (Figure 3c), in which the coarsest fractions in the range of 0.6–1 mm were cut successively, in contrast to the previous cases, deterioration of the rheological properties of the granulates was observed along with the removal of subsequent fractions, as evidenced by the recorded courses of the flow function of granulates in relation to the baseline value (SG).

The flow function of the production granulate (PG) shows a much better flow of this material than the soft waste granulate (SG), and its course places this material on the border of the easy-flowing and free-flowing area. Changes in the particle size composition to a lesser extent than in the case of SG granulate affect the changes in the flowability of the granulate.

In the case of series 1 (Figure 4a), in which the finest fractions were cut off, similarly to the granulates from soft waste, an improvement in the flowability of the granulates was observed. All tested samples from this series were below the course of the flow function of the production granulate (PG), entering the free-flowing area, while the registered changes were smaller than in the case of SG granulate.

Series 2 (Figure 4b), for which the fractions were cut off from the bottom while simultaneously removing the coarsest fractions, showed a different relationship than that observed for SG granulate. A significant deterioration of the flow was observed for samples 8 and 9, from which fine fractions 0-0.18 mm and above 1 mm were removed (sample 8 even entered the cohesive area). For samples 10 and 11, from which fractions 0-0.30 mm and above 0.7 mm were removed, an increase in flowability was observed, and the course of the flow function for these samples entered the free-flowing area.

In the case of series 3 (Figure 4c), where the coarsest fractions were cut off, no significant changes in the flow of granulates were observed, and the course of the flow function for samples from this series practically coincided with the course of the function recorded for the production granulate PG.

### 3.3. Bulk Density

**Figure 5:**
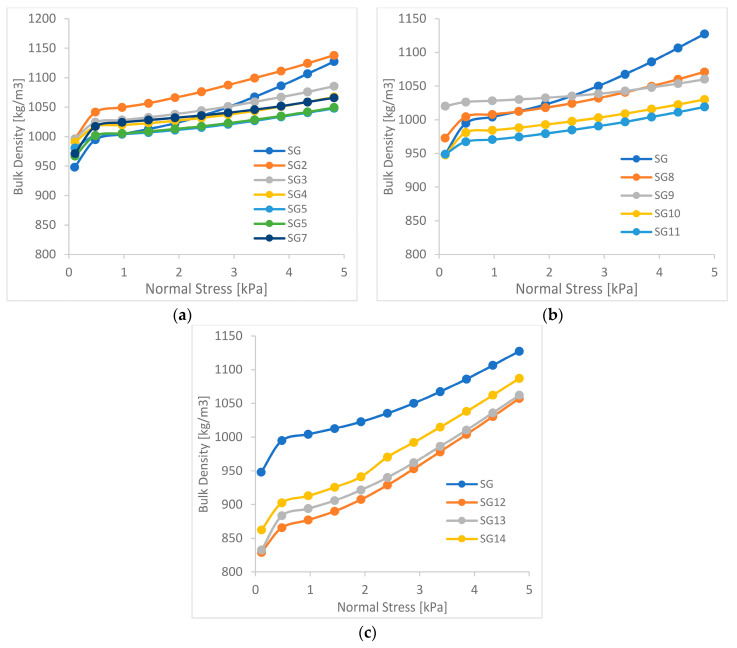
Bulk density of soft waste granulates (**a**) series 1; (**b**) series 2; (**c**) series 3.

**Figure 6:**
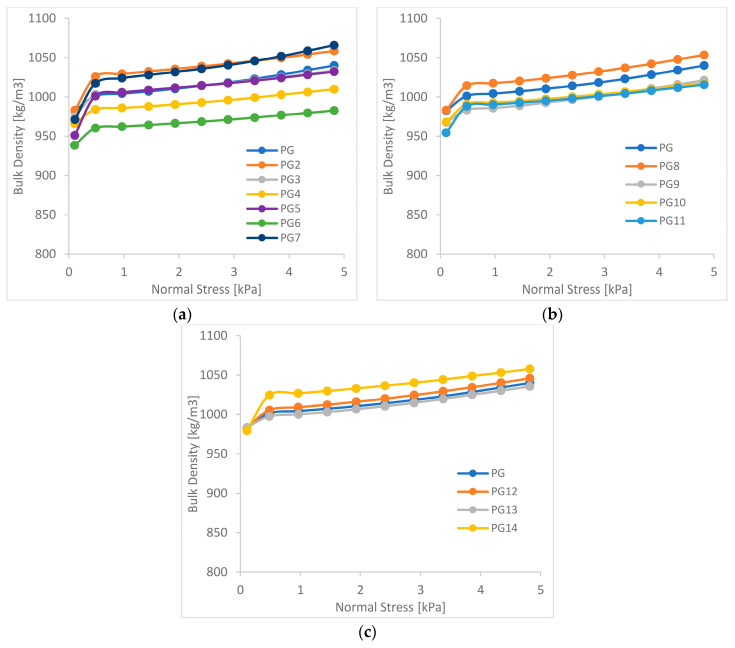
Bulk density of production granulates (**a**) series 1; (**b**) series 2; (**c**) series 3.

The results of study on changes in bulk density as a function of consolidation pressure are presented in Figure 5 and Figure 6.

The courses of changes in the bulk density as a function of consolidation stress confirm the differences visible in the results of rheological measurements. The production granulates changed their flow character, to a much lesser extent than the granulates from soft waste, with the subtraction of individual fractions. In the case of granulates from soft waste in series 1 (Figure 5a) and 2 (Figure 5b), where the bulk density graphs showed an improvement in rheological properties, smaller increases in density were observed with an increase in consolidation stress. In series 3 (Figure 5c), for which deterioration of rheological properties was observed, the courses of density changes showed significantly greater increases in the bulk density with the increase in consolidation stress, which is characteristic of less flowable materials [15,16].

For the production granulate, no significant changes in the increase in bulk density were observed for each series, which corresponds to the course of the flow function of this material, for which the changes in parameters were much smaller [17,18].

## 4. Conclusions

Differences between granulates from soft waste and production granulates related to the morphology of grains and granulometric composition translate into their rheological properties. Classic standard tests, consisting of measuring the angle of fall, only to a small extent illustrate these differences, and the measurements of the flow function are a much better analytical tool. The recorded curves not only show the differences between the soft waste and production granulates, but also precisely illustrate the changes in the flow of granulates along with the change in their particle size distribution.

The results of the conducted research show that the relatively inexpensive technological operation consisting in separating the finest (0–0.125 mm) and the coarsest (2–1 mm) fractions from soft waste granulates, significantly improves the rheological properties of this granulate, bringing them closer to the parameters characterized by the production granulate.

Such granulate can be reused in production as an additive to production granulate or used as an additional layer in new products, which leads to a significant reduction in production costs, and, equally important, a significant reduction in energy consumption, and thus reduction of CO_2_ emissions.

## Figures and Tables

**Table 1:** Particle size distribution of soft waste granulates (SG), mass %.

Range [mm]	Granulates													
	SG	SG2	SG3	SG4	SG5	SG6	SG7	SG8	SG9	SG10	SG11	SG12	GS13	SG14
2.00–1.00	25.5	31.0	34.2	37.6	43.0	47.3	53.3	0	0	0	0	0	0	0
1.00–0.71	10.9	13.3	14.7	16.1	18.5	20.3	22.9	19.3	22.3	25.8	0	0	0	14.7
0.71–0.60	4.2	5.1	5.6	6.1	7.0	7.7	8.7	7.3	8.5	9.8	18.3	0	6.5	5.6
0.60–0.50	7.2	8.8	9.7	10.6	12.2	13.4	15.1	12.7	14.7	17.0	31.6	12.1	11.3	9.7
0.50–0.425	6.1	7.4	8.1	8.9	10.2	11.3	0	10.7	12.3	14.3	26.6	10.2	9.5	8.1
0.425–0.30	5.4	6.5	7.2	7.9	9.1	0	0	9.5	10.9	12.6	23.5	9.0	8.4	7.2
0.30–0.25	8.7	10.5	11.6	12.8	0	0	0	15.3	17.6	20.4	0	14.6	13.6	11.6
0.25–0.18	6.7	8.2	9.0	0	0	0	0	11.8	13.7	0	0	11.3	10.6	9.0
0.18–0.125	7.6	9.2	0	0	0	0	0	13.4	0	0	0	12.8	12.0	10.2
<0.125	17.8	0	0	0	0	0	0	0	0	0	0	30.0	28.0	23.9

**Table 2:** Particle size distribution of production granulates (PG), mass %.

Range [mm]	Granulates													
	PG	PG2	PG3	PG4	PG5	PG6	PG7	PG8	PG9	PG10	PG11	PG12	PG13	PG14
2.00–1.00	1.9	1.9	2.0	2.1	2.3	3.0	3.7	0	0	0	0	0	0	0
1.00–0.71	18.4	18.6	19.3	20.7	22.1	29.0	35.7	19.0	19.7	21.1	0	0	0	18.8
0.71–0.60	10.8	11.0	11.4	12.2	13.0	17.0	21.0	11.1	11.6	12.4	17.1	0	13.6	11.0
0.60–0.50	20.4	20.7	21.4	23.0	24.5	32.1	39.6	21.1	21.8	23.4	32.3	30.0	25.6	20.8
0.50–0.425	12	12.2	12.6	13.5	14.4	18.9	0	12.4	12.8	13.8	19.0	17.6	15.1	12.2
0.425–0.30	19.9	20.1	20.8	22.3	23.8	0	0	20.5	21.3	22.9	31.5	29.3	25.0	20.3
0.30–0.25	5.5	5.5	5.7	6.1	0	0	0	5.7	5.9	6.3	0	8.1	6.9	5.6
0.25–0.18	6.4	6.5	6.8	0	0	0	0	6.6	6.9	0	0	9.4	8.0	6.5
0.18–0.125	3.5	3.5	0	0	0	0	0	3.6	0	0	0	5.1	4.4	3.6
<0.125	1.2	0	0	0	0	0	0	0	0	0	0	1.8	1.5	1.2

**Table 3:** Bulk density and angle of fall.

	Granulates													
	SG	SG2	SG3	SG4	SG5	SG6	SG7	SG8	SG9	SG10	SG11	S12	SG13	SG14
Bulk density (g/cm^3^)	0.97	1.03	1.02	1.01	1.02	1.00	1.00	0.99	0.98	0.97	0.96	0.84	0.87	0.9
Angle of fall (°)	31	28	29	28	28	27	28	29	28	28	28	34	33	32
							Granulates							
	PG	PG2	PG3	PG4	PG5	PG6	PG7	PG8	PG9	PG10	PG11	PG12	PG13	P14
Bulk density (g/cm^3^)	1.04	1.04	1.02	1.01	1.01	0.98	0.97	1.04	1.02	1.04	1.00	1.04	1.03	1.03
Angle of fall (°)	24	22	22	22	22	22	22	22	23	22	22	23	23	23

## Data Availability

Not applicable.
